# Circulating Th1 and Th2 Subset Accumulation Kinetics in Septic Patients with Distinct Infection Sites: Pulmonary versus Nonpulmonary

**DOI:** 10.1155/2020/8032806

**Published:** 2020-09-14

**Authors:** Ming Xue, Yuying Tang, Xu Liu, Mingyuan Gu, Jianfeng Xie, Ling Liu, Yingzi Huang, Fengmei Guo, Yi Yang, Haibo Qiu

**Affiliations:** Department of Critical Care Medicine, Zhongda Hospital, School of Medicine, Southeast University, Nanjing 210009, China

## Abstract

**Background:**

Persistent peripheral CD4^+^T cell differentiation towards T helper (Th)2 rather than Th1 has been proved to be related to immunosuppression and poor prognosis in sepsis. However, it is unclear whether these circulating Th1 and Th2 subtype accumulations differed in septic populations of distinct infection sites and presented different associations with outcomes among patients with pulmonary versus nonpulmonary sepsis.

**Methods:**

From a secondary analysis of a prospective observational study, seventy-four previously immunocompetent patients with community-acquired severe sepsis within 24 hours upon onset were enrolled. Whole blood was collected on the admission day (D0), 3rd day (D3), and 7th day (D7). The patients were classified as pulmonary (*n* = 52) and nonpulmonary sepsis (*n* = 22). Circulating Th1 and Th2 populations were evaluated by flow cytometry, and clinical data related to disease severity and inflammatory response were collected. The associations of circulating Th1 and Th2 subset accumulations with distinct infection sites or outcomes within subgroups were explored.

**Results:**

Patients with pulmonary sepsis held similar disease severity and 28-day mortality with those of nonpulmonary sepsis. Of note is the finding that circulating Th2 levels on D7 (*P* = 0.04) as well as Th2/Th1 on D3 (*P* = 0.01) and D7 (*P* = 0.04) were higher in the pulmonary sepsis compared with nonpulmonary sepsis while Th1 levels were lower on D0, D3, and D7 (*P* = 0.01, <0.01, and =0.05, respectively). Compared to 28-day survivors, higher Th2/Th1 driven by increased Th2 were observed among 28-day nonsurvivors on D3 and D7 in both groups. The association between circulatory Th2 populations or Th2/Th1 and 28-day death was detected in pulmonary sepsis (*P* < 0.05, HR > 1), rather than nonpulmonary sepsis.

**Conclusions:**

Circulating Th2 accumulation was more apparent among pulmonary sepsis while nonpulmonary sepsis was characterized with the hyperactive circulating Th1 subset among previously immunocompetent patients. This finding suggested that circulating Th1 and Th2 subset accumulations vary in septic subgroups with different infection sites.

## 1. Introduction

Sepsis remains a major healthcare problem worldwide accounting for a high number of deaths every year [1–4]. Numerous findings of immunomodulatory therapy showing benefit in survival and organ protection in preclinical models of sepsis ended up with failures in translation to clinical treatment [5]. One of the underlying explanations is the heterogeneity among septic population in current studies, including the organism and site of infection as well as the host response diversity with the varying confounders [5–11].

The infection site is thought to impact the clinical outcomes for several potential mechanisms, including the pathogens' nature more likely to be at the certain sites and the following life-threatened organ failures, such as severe hypoxemia [9, 11, 12]. Sepsis is the result of host immune response to infection, and the original site of infection might be the main driver of secondary organ injury or even death. A prospective longitudinal study recently published [13] demonstrated notable differences in baseline predisposition, host responses, and clinical outcomes by site of infection during sepsis. Phenotypic heterogeneity was partly induced by the site of infection during sepsis. The clarification of the underlying impact on immunological alterations by different subtypes will assist better precise interventions and design future clinical trials.

Sites of primary infection are reported with no association with mHLA-DR expression kinetics in patients with septic shock [14]. However, the relevance of infection sites with T cell-mediated immune response, which worked as one of potentially pathobiology-driven septic endotypes [6], lacks evaluation. There have been studies reporting a decreased absolute count and increased apoptosis of CD4 and CD8 lymphocytes during sepsis with different types of underlying infection [12]. Our previous study showed that patients with delayed or no improvement in a Th1/Th2 skewing with Th2 predominate have a higher risk for poor outcomes compared with patients without [15]. Importantly, if circulating Th1 or Th2 subtype accumulation varies according to the infection sites, this may aid clinicians in better understanding the endotype signature of T cell immune response imbalance and exploring potential targets among appropriate populations as currently septic patients with different infection sources are often mixed in most studies [16, 17].

Th1/Th2 skewing upon sepsis with Th2 dominance and its persistent existence has been proved to be associated with poor outcome in severe sepsis [15]. With these data, targeting a relatively homogeneous population, of which all were previously immunocompetent patients with new-onset community-acquired severe sepsis, we performed a second analysis and classified the patients by distinct infection sites: pulmonary vs. nonpulmonary. The objective was to explore whether the kinetics of circulating Th1 and Th2 subtype accumulations differed and their relation to clinical outcomes stratified by infection sites during sepsis.

## 2. Methods

This study is a second analysis from a single-centre, prospective, and observational study. The original study was conducted in accordance with the amended Declaration of Helsinki. The full protocol approved by the Institutional Ethics Committee of Zhongda Hospital (Approval Number: 2014ZDSYLL086) and registered with ClinicalTrials.gov (NCT02883218) could be referred to the original paper [15].

### 2.1. Study Population

**Figure 1:**
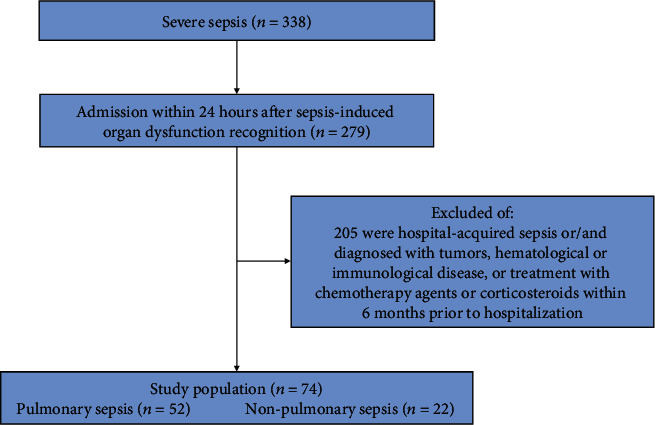
Study population. Diagnostic procedure was up to the treating clinicians according to the criteria of the American College of Chest Physicians/Society of Critical Care Medicine.

The inclusion and exclusion criteria have been described previously [15]. Of 338 patients admitted with a diagnosis of community-acquired severe sepsis according to the criteria of the American College of Chest Physicians/Society of Critical Care Medicine [18] between September 18, 2014, and September 28, 2016 (Figure 1), 71 patients were included in the original analysis after 3 were excluded because Th1 and Th2 measurements on D0, D3, and D7 could not be obtained [15]. In this present study, our primary interest was to investigate whether circulatory T helper subtype accumulations differed and their relation to clinical outcomes; thus, all these 74 patients were included. Among the study population, 52 patients were classified as pulmonary sepsis while 22 were as nonpulmonary sepsis according to the clinically suspected infection sites.

### 2.2. Data Collection

Demographic and clinical information including systemic inflammatory indicators, such as temperatures, heart rates, and count of white blood cell (WBC), as well as absolute lymphocyte count (ALC), procalcitonin (PCT), and hypersensitive C-reactive protein (hs-CRP) on D0, D3, and D7, and outcomes including occurrence of complicated organ dysfunction and prognosis within 28 days were collected from medical documents as described previously [15]. Definitions of organ failures were referred to previous descriptions [19–23]. Whole blood was collected on D0, D3, and D7 for T lymphocyte subpopulation measurement by flow cytometry and supernatant cytokine detection of interferon- (IFN-) *γ*, interleukin- (IL-) 2, IL-4, IL-6, and IL-10 by enzyme-linked immunosorbent assay (ELISA) as described previously [15].

### 2.3. Statistical Analysis

Th1 and Th2 populations were expressed as numbers in CD3^+^CD8^−^T lymphocytes (%). Data were analysed using SPSS Version 23 (IBM, Chicago, IL, USA) and GraphPad Prism Version 5.3 (San Diego, CA, USA). Descriptive statistics, including the mean ± standard deviation (SD) and median (interquartile range (IQR) defined as the 25th and 75th percentiles), were used as appropriate. Comparisons between pulmonary sepsis and nonpulmonary sepsis or between subgroups according to 28-day prognosis were performed using unpaired *t*-tests, Mann-Whitney *U* tests, or chi-squared tests, as appropriate. A power calculation for the detected difference in terms of CD4^+^T cell subpopulations was performed by the Power Analysis and Sample Size software (PASS 2008. Citation: Hintze J (2008). NCSS, LLC. Kaysville, Utah, USA). Kaplan-Meier analysis was performed to determine the survival lifetimes between the pulmonary and nonpulmonary sepsis for 28-day survival, and a log-rank test was used to compare curves. All tests were two-tailed, and a value of *P* < 0.05 was considered statistically significant.

Univariate analysis of Cox regression in the pulmonary sepsis cohort (*n* = 52) or the nonpulmonary sepsis cohort (*n* = 22) was performed respectively, using SPSS, to identify variables that were independently associated with 28-day mortality. The variables included demographics, severity score, and inflammatory and immune indicators including WBC, ALC, Th1 and Th2 populations, and PCT as well as hs-CRP and alterations of these indicators. Variables identified with a threshold of *P* < 0.05 were investigated for the associations with 28-day mortality in a Cox proportional hazards model. Specific Cox models were conducted for each variable that was mathematically coupled or collinear with each other, such as WBC and ALC on D0, D3, and D7, respectively. Hazard ratios were calculated for each variable included in the final model with 95% confidence intervals (CIs).

## 3. Results

### 3.1. Subject Characteristics, Clinical Presentation, and Outcomes

Patients with pulmonary sepsis and nonpulmonary sepsis had similar distribution in age, gender, aetiology, and similar alcohol use and smoke history (Table 1). Chronic cardiac dysfunction was more common in patients with pulmonary sepsis, compared to those with nonpulmonary sepsis (44.2% vs. 18.2%, *P* = 0.04), whereas there were no significant differences in preexisting hypertension, diabetes, cerebrovascular disease, or chronic renal dysfunction.

**Figure 2:**
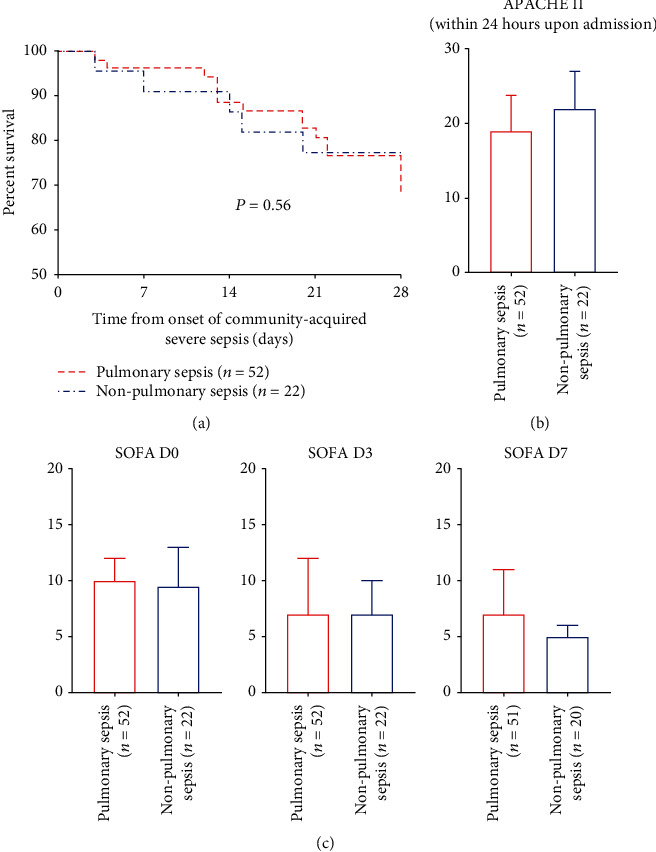
Survival curves within 28 days upon admission (a) and disease severity according to the APACHE II upon admission (b) and SOFA scores on D0, D3, and D7 (c) showed no significant difference between pulmonary and nonpulmonary sepsis. APACHE = Acute Physiology and Chronic Health Evaluation; SOFA = Sepsis-related Organ Failure Assessment.

The 28-day mortality in pulmonary and nonpulmonary sepsis was 30.8% and 22.7% with no significant difference in the survival curve (Figure 2(a)). Disease severity was comparable according to the Acute Physiology and Chronic Health Evaluation (APACHE) II and Sepsis-related Organ Failure Assessment (SOFA) scores (Table 1 and Figures 2(b) and 2(c)). In the pulmonary sepsis cohort, 28-day survivors had lower APACHE II upon admission (*P* = 0.02) and SOFA scores on day 3 and day 7 (*P* = 0.02 and 0.01) compared with nonsurvivors. In nonpulmonary sepsis, no difference was observed in age and gender distribution, documented comorbidities, smoke and alcohol use history, and disease severity upon admission as well as on D3 and D7 (Table 1). Compared to nonpulmonary sepsis, the pulmonary sepsis group was demonstrated with a higher risk of respiratory failure (OR 1.136, 95% CI 0.958-1.347, Table 2). In addition, the odds ratio of complications with more organ dysfunctions in pulmonary sepsis to nonpulmonary sepsis kept increasing, whereas no significant difference was detected within the present study.

### 3.2. Inflammatory and Immune Indicators in the Pulmonary versus Nonpulmonary Cohorts

**Figure 3:**
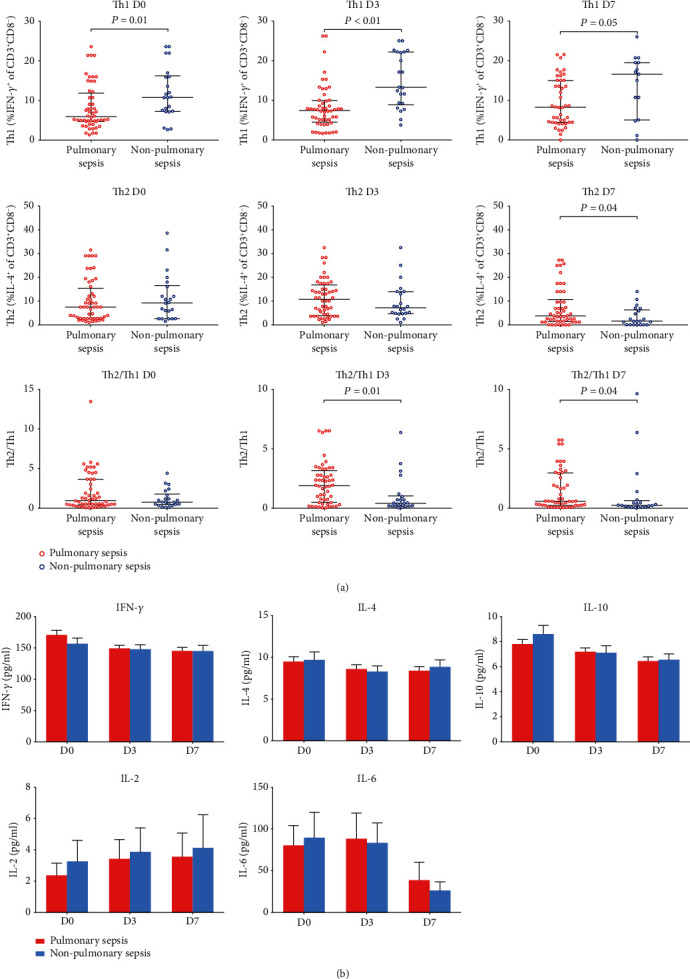
Circulating Th1 and Th2 subset accumulations and related cytokine levels of IFN-*γ*, IL-2, IL-4, IL-6, and IL-10 in pulmonary and nonpulmonary sepsis. Data was presented by scatter dot plots and lines of median with the interquartile range. *P* values shown were determined by using the Mann-Whitney *U* test.

Compared to those with nonpulmonary sepsis, patients with pulmonary sepsis held lower Th1 population on D0 (*P* = 0.01), D3(*P* < 0.01), and D7 (*P* = 0.05) and higher T helper 2 cell population on D7 (*P* = 0.04), leading to the significant difference of Th2/Th1 observed on D3 (*P* = 0.02) and D7 (*P* = 0.04) (Figure 3), while Th1- or Th2-related cytokine levels of IFN-*γ*, IL-2, IL-4, and IL-10 as well as other inflammatory indicators, including IL-6, ALC, WBC, hs-CRP, and PCT, were comparable in the two groups (Figure 3 and Supplementary Fig S1).

### 3.3. Associations of Th1 and Th2 Subset Accumulations with 28-Day Prognosis in Subgroups by Infection Sites

**Figure 4:**
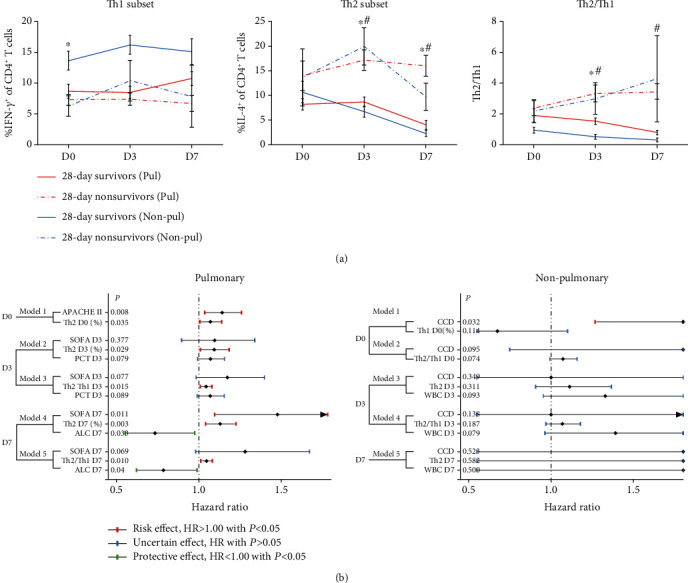
Kinetics of Th1, Th2, and Th2/Th1 stratified by 28-day outcomes and Cox proportional hazards models in pulmonary and nonpulmonary sepsis. (a) Kinetics of Th1, Th2, and Th2/Th1 stratified by the 28-day outcome on D0, D3, and D7 in pulmonary and nonpulmonary sepsis. Data was presented as mean with mean squared error; *P* values shown were determined by using the Mann-Whitney *U* test: ^∗^*P* < 0.05 between 28-day survivors and nonsurvivors within the pulmonary sepsis group, ^#^*P* < 0.05 between 28-day survivors and nonsurvivors within the nonpulmonary sepsis group. (b) Death hazard ratios for severity scores, peripheral blood cell count, and T helper population within the study period in distinct subgroups. Significant risk factors are in red; factors without significance are in blue; significant protective factors are in green. APACHE = Acute Physiology and Chronic Health Evaluation; CCD = chronic cardiac dysfunction; SOFA = Sepsis-related Organ Failure Assessment; WBC = white blood cell; ALC = absolute lymphocyte count.

As demonstrated in Figure 4, septic patients who survived within 28 days in both the pulmonary and nonpulmonary groups held lower Th2 frequency along with lower Th2/Th1 on D3 and D7, among which the significance of the Th2/Th1 difference on D7 between groups stratified by the 28-day outcome in the nonpulmonary group was lacking mainly due to the rather small number of 28-day nonsurvivors (*n* = 3). Univariate regression (Supplementary Table S1 and S2) and Cox models for 28-day mortality were applied in the pulmonary and nonpulmonary subgroups, respectively, to further determine T helper subsets' contribution to the 28-day outcome. In the pulmonary sepsis group, Th2 populations on D0, D3, and D7; Th2/Th1 on D3; and disease severity scores including APACHE II within 24 hours upon admission and SOFA on D7 were independently associated with increased risk of death within 28 days (*P* < 0.05, hazard ratio (HR) > 1, Figure 4(b)), while ALC on D7 was independently associated with reduced mortality (*P* < 0.05, HR < 1, Figure 4(b)). In the nonpulmonary sepsis group, no significant association of T helper subpopulations with 28-day prognosis was detected when combined with multiple factors such as comorbidities and disease severity.

## 4. Discussion

The objective of our study was to determine whether Th1 and Th2 subtype accumulation over time upon sepsis onset and their associations to clinical outcomes differed between pulmonary and nonpulmonary sepsis. In the present study, patients with pulmonary and nonpulmonary sepsis held the similar levels in numbers of organ failures, illness severity, and 28-day mortality. Notable differences were observed between pulmonary and nonpulmonary sepsis in terms of Th1 and Th2 cells accumulation and its association with 28-day prognosis. Th2 accumulation gradually appeared obvious in one week after severe sepsis onset in pulmonary sepsis while Th1 populations were higher in nonpulmonary sepsis. Th2 accumulation and the increase of Th2/Th1 were independently associated with increased risk of 28-day death only in the pulmonary sepsis but not in the nonpulmonary sepsis though Th2 subset accumulations were detected more apparently among 28-day nonsurvivors in both groups. To our knowledge, this study shows for the first time the distinct Th1 and Th2 subtype accumulations with their associations with 28-day prognosis in patients with pulmonary sepsis versus nonpulmonary sepsis. These data will help clinicians to better understand the CD4^+^T cell immune response imbalance among the heterogeneous septic population and illuminate important aspects of therapeutically targeting populations.

Whether the infection site impacts clinic outcomes still remains controversial. A significant association between the site of infection and the inhospital mortality in patients with sepsis or septic shock was reported in a secondary analysis of a multicentre prospective cohort study recently [24], while several studies showed that the evidence for the conclusive statement about the role of the infection site in mortality among sepsis might not be robust for heterogeneity of study population by the present literature [25, 26]. However, the impact of the infection site on patterns of organ failures might differ. Neurological dysfunction was reported to be more common in respiratory infection than others while the abdominal infections led to a higher incidence of circulatory failure than others did [25], with both of which our results were consistent with the same trends in comparisons between pulmonary and nonpulmonary sepsis. Of note, the impacts of antimicrobial therapy or organ support capacities in different medical centres have been largely neglected in most studies, which might cover the inherent relationship between infection sites and clinic outcomes.

Peripheral Th1 and Th2 populations, as parts of CD4^+^T cell-mediated adaptive immunity, drive and control immune responses. Th1 cells are proinflammatory by releasing cytokine interferon- (IFN-) *γ* for phagocytosis and intracellular killing of microbes while Th2 cells are anti-inflammatory by secreting the mainly anti-inflammatory cytokines interleukin- (IL-) 10 and IL-4. Persistent Th2 dominance in the balance of Th1 and Th2 was demonstrated with a significant association with 28-day mortality among the mixed septic population in the previous analysis of the present study cohort [15], and we further proposed that Th1 and Th2 subtype accumulations vary in distinct subpopulations of infection sources. Our results confirmed the hypothesis and showed that pulmonary sepsis presented Th2 dominance while nonpulmonary sepsis was characterized with Th1 subset dominance. The current results partly supported the findings of notable differences in host responses by the site of infection during sepsis in Stortz et al.'s study [13], in which abdominal infections experienced robust proinflammation, partly in line with our findings of proinflammatory Th1 predominance in the nonpulmonary sepsis, while immunosuppression biomarkers of pulmonary sepsis normalized faster which was likely due to the distinct resources of hospital-acquired pneumonia in Stortz et al.'s study and community-acquired severe sepsis in our study.

Several potential mechanisms for the difference of Th1 and Th2-CD4^+^T subset accumulation between pulmonary and nonpulmonary sepsis might exist. First, certain anatomical sites differed in immune response characteristics. Previous studies suggested that Th2 cells might be particularly adapted for migration to lungs which are thought to host Th2-type inflammation while Th1 cells were more readily recruited to the peritoneal cavity and likely to induce a systematic inflammatory response, which might be consistent with more common complications of circulatory failure [27–29]. Second, some certain anatomical sites may be related to one or more highly virulent pathogens than other sites, leading to distinct cell-mediated immune response [30, 31]. Th2 cells would dominate in affected sites under conditions of parasitic, viral, or atypical organism infection while Th1 cells are more involved in the bacterial infections [31–34]. Even if most of the included cases were clinically suspected undocumented infection, viral or other atypical organism infections are more common in the respiratory tract than other sites for its sensitive and functional mucosal tissue [35]. In addition, the absent significance of Th2 accumulation's association with 28-day death in the nonpulmonary sepsis cohort might be partly due to a relatively small sample size in the present setting and it might be attenuated by other factors, including the comorbidities, disease severity, and the implementation of appropriate early antibiotics.

Our study has several strengths that focus on a more homogeneous population and appropriate core outcomes to get a relatively robust conclusion. By enrolling previously immunocompetent community-acquired severe sepsis patients without past histories affecting the immune system and with recordable manifestation or laboratory findings of sepsis-induced organ dysfunction within 24 hours, we tried to guarantee enrolment in relatively the same and early stage of the septic process as possible. What is more, CD4^+^T cell subtype accumulation and Th1 and Th2 population accumulation in response to severe sepsis and secondary organ injuries, rather than the prognosis, were set as prior outcomes, which might closely reflect the host response and its heterogeneity to the original infection in the acute phase.

There are several potential limitations. First, all diagnostic and treatment decisions were up to the treating physician, which might result in cases with unidentified focus and lack of proven microbiological aetiology. Second, the sample size included was small. The relatively low percentage of nonpulmonary to that of pulmonary might bring potential bias though the component of these two distinct populations was consistent with the latest published national cross-sectional survey of sepsis epidemiology in Chinese ICUs [36]. Besides, even if we performed the power calculation of the detected difference lying in the frequency of the Th1 and Th2 subsets or Th2/Th1 values within two groups (Table S3), the limited sample size obtained in one medical centre might degrade the power. Third, the causation between Th1 or Th2 subset accumulation and infection sites cannot be confirmed with the second analysis of an observational study. Additionally, our data focused on the alterations of peripheral Th1 and Th2 populations within one week upon sepsis onset. Th2 and Th1 populations at the cellular level could only reflect a part of CD4^+^T cell immune response to the infection. Sepsis-induced adaptive immune dysfunction should be evaluated comprehensively, including other immune cell types like Th17 and multilevels in transcription and protein as well as the function of these immune cells, not limited to the number or frequency. Given the recent study by Stortz et al. [13], the impact on host response by sepsis is site-dependent and may occur later in the clinical course. Future studies need to be done at multiple levels of immunological behaviours for long-term outcomes.

## 5. Conclusions

In conclusion, the present study reports distinct circulating Th1 and Th2 subset accumulations between pulmonary and nonpulmonary sepsis. Despite of the similar levels of inflammatory indicators, disease severity, and mortality, accumulation of Th2 was more apparent in pulmonary sepsis while hyperactive Th1 was in nonpulmonary sepsis. This study provides new evidence for the varying Th1 and Th2 subsets of CD4^+^T cell immune response in septic subgroups by infection sites, which will help further understand the endotype variations among heterogeneous sepsis and select the appropriate population when proposing T cell-targeted therapy in the future.

## Figures and Tables

**Table 1:** Demographic and clinical characteristics of patients with pulmonary and nonpulmonary sepsis.

	Pulmonary sepsis (*n* = 52)				Nonpulmonary sepsis (*n* = 22)				*P* (pulmonary vs. nonpulmonary)
	Overall *n* = 52	28-day survivor *n* = 36 (69.2%)	28-day nonsurvivor *n* = 16 (30.8%)	*P*	Overall *n* = 22	28-day survivor *n* = 17 (77.3%)	28-day nonsurvivor *n* = 5 (22.7%)	*P*	
Age (years)	74 ± 12	75 ± 13	69 ± 10	0.04	67 ± 21	65 ± 23	77 ± 10	0.39	0.30
Gender (male/female)	34/18	25/11	9/7	0.53	13/9	12/5	1/4	0.12	0.79
APACHE II (mean ± SD)	20 ± 6	19 ± 5	23 ± 6	0.02	22 ± 6	21 ± 6	24 ± 6	0.37	0.19
SOFA (mean ± SD)									
D0	10 ± 3	9 ± 3	11 ± 3	0.18	10 ± 3	10 ± 3	10 ± 3	0.99	0.99
D3	8 ± 4	7 ± 4	10 ± 4	0.02	8 ± 3	8 ± 2	9 ± 5	0.65	0.91
D7	7 ± 4	7 ± 3	10 ± 3	0.01	6 ± 2	6 ± 2	7 ± 4	0.18	0.19
Aetiology %									
Bacteria	19 (36.5)	11 (30.6)	8 (50)	0.22	13 (59.1)	10 (58.8)	3 (60)	>0.99	0.63
Virus	4 (7.7)	2 (5.6)	2 (12.5)	0.58	1 (4.5)	1 (5.9)	0 (0)	>0.99	>0.99
Others or undetected	29 (55.8)	23 (63.9)	6 (37.5)	0.13	7 (31.8)	6 (35.3)	1 (20)	>0.99	0.20
Comorbidities (*n* (%))									
Hypertension	29 (55.8)	21 (58.3)	8 (50)	0.76	10 (45.5)	7 (41.2)	4 (80)	0.31	0.80
Diabetes	14 (26.9)	9 (25)	5 (32)	0.74	6 (27.3)	4 (23.5)	2 (40)	0.59	>0.99
Chronic cardiac dysfunction	23 (44.2)	18 (50)	5 (32)	0.24	4 (18.2)	2 (11.8)	2 (40)	0.21	0.04
Cerebrovascular disease	23 (44.2)	18 (50)	5 (32)	0.24	5 (22.7)	4 (23.5)	1 (20)	>0.99	0.12
Chronic renal dysfunction	4 (7.7)	4 (11.1)	0 (0)	0.30	1 (4.5)	0 (0)	1 (20)	0.23	>0.99
Smoke history				>0.99				>0.99	0.83
Never	36 (69.2)	24 (66.7)	12 (75)		13 (59.1)	9 (52.9)	5 (100)		
Previous (stopped >3 months)	11 (21.2)	8 (22.2)	3 (18.75)		3 (13.6)	3 (17.6)	0 (0)		
Former (stopped ≤3 months)	0 (0)	0 (0)	0 (0)		0 (0)	0 (0)	0 (0)		
Current	5 (9.6)	4 (11.1)	1 (6.25)		5 (22.7)	5 (11.1)	0 (0)		
Alcohol use (of pure alcohol)				>0.99				>0.99	0.67
Never or daily intake ≤ 50 g	46 (88.5)	32 (88.9)	16 (100)		19 (86.4)	15 (88.2)	5 (100)		
Previous (daily intake >, stopped >3 months)	2 (3.8)	2 (5.6)	0 (0)		0 (0)	0 (0)	0 (0)		
Former (daily intake >, stopped ≤3 months)	0 (0)	0 (0)	0 (0)		0 (0)	0 (0)	0 (0)		
Current intake (>50 g)	2 (3.8)	2 (5.6)	0 (0)		2 (9.1)	2 (11.8)	0 (0)		

Continuous variables are presented as mean ± standard deviation and compared by using the Mann-Whitney *U* test. Categorical data are presented as no. (%) and compared by using the Pearson *χ*^2^ test. The number of organ failures includes only nonpulmonary organ failures. APACHE = Acute Physiology and Chronic Health Evaluation; SD = standard deviation; SOFA = Sepsis-related Organ Failure Assessment.

**Table 2:** Complicated organ dysfunctions within 28 days in pulmonary and nonpulmonary sepsis.

	Nonpulmonary sepsis (*n* = 22)	Pulmonary sepsis (*n* = 52)	*P*, OR (95% CI) (pulmonary to nonpulmonary)
Complicated with			
Respiratory failure, *n* (%)	19 (86.4)	51 (98.1)	0.042, 1.136 (0.958-1.347)
Circulatory shock, *n* (%)	20 (91.0)	46 (88.5)	0.757, 0.973 (0.825-1.147)
AKI, *n* (%)	8 (36.4)	22 (42.3)	0.634, 1.163 (0.615-2.201)
AGI, *n* (%)	4 (18.2)	20 (38.5)	0.089, 2.115 (0.817-5.474)
CNS dysfunction, *n* (%)	2 (9.1)	13 (25)	0.120, 2.750 (0.676-11.183)
No. of organ failures (total)			
Median (IQR)	2 (2, 3)	3 (2, 4)	0.057
≥2, *n* (%)	18 (86.4)	49 (94.2)	0.095, 1.152 (0.935-1.418)
≥3, *n* (%)	9 (40.9)	33 (63.5)	0.073, 1.551 (0.901-2.670)
≥4, *n* (%)	2 (9.1)	14 (26.9)	0.089, 2.962 (0.734-11.705)
≥5, *n* (%)	0 (0)	3 (5.8)	0.250, -
No. of organ failures (excluding lung-respiratory failure)			
≥1, *n* (%)	20 (90.9)	50 (96.2)	0.362, 1.058 (0.917-1.220)
≥2, *n* (%)	9 (40.9)	33 (63.5)	0.073, 1.551 (0.901-2.670)
≥3, *n* (%)	2 (9.1)	14 (26.9)	0.089, 2.962 (0.734-11.705)
≥4, *n* (%)	0 (0)	3 (5.8)	0.250, -

OR = odds ratio; AKI = acute kidney injury; AGI = acute gastrointestinal injury; CNS = central nervous system; IQR = interquartile range.

## Data Availability

All data generated and/or analysed during this study are included in this published article and its supplementary information files.

## Supplementary Materials

Supplementary Materials Table S1: univariate Cox regression analysis of variables associated with the 28-day mortality in pulmonary sepsis. Table S2: univariate Cox regression analysis of variables associated with the 28-day mortality in nonpulmonary sepsis. Table S3: the power calculation of the parameter with detected difference between pulmonary and nonpulmonary sepsis with *α* = 0.10. Figure S1: inflammatory and immune indicators in pulmonary and nonpulmonary sepsis.
