# Supplementary material for: Circulating Th1 and Th2 Subset Accumulation Kinetics in Septic Patients with Distinct Infection Sites: Pulmonary versus Nonpulmonary
Source: Mediators Inflamm. 2020 Sep 14;2020:8032806. doi: 10.1155/2020/8032806 (PMC7509553; doi:10.1155/2020/8032806)
Supplement: Supplementary Materials — Table S1: univariate Cox regression analysis of variables associated with the 28-day mortality in pulmonary sepsis. Table S2: univariate Cox regression analysis of variables associated with the 28-day mortality in nonpulmonary sepsis. Table S3: the power calculation of the parameter with detected difference between pulmonary and nonpulmonary sepsis with α = 0.10. Figure S1: inflammatory and immune indicators in pulmonary and nonpulmonary sepsis. [file 8032806.f1.docx]

**Table S1** Univariate cox regression analysis of variables associated with the 28-day mortality in pulmonary sepsis.

| **Variables** | **P** | **Exp(B)** |
| --- | --- | --- |
| Age | 0.18 | 0.97 |
| gender | 0.39 | 1.54 |
| Comorbidities |  |  |
| Hypertension | 0.44 | 0.68 |
| Cardiovascular dysfunction | 0.19 | 0.49 |
| Diabetes | 0.77 | 1.77 |
| Cerebrovascular disease | 0.25 | 0.54 |
| Chronic renal dysfunction | 0.41 | 0.43 |
| Severity score |  |  |
| **APACHE II** | **0.02** | **1.12** |
| SOFA D0 | 0.20 | 1.12 |
| **SOFA D3** | **0.02** | **1.19** |
| **SOFA D7** | **0.01** | **1.26** |
| P/F ratio | 0.69 | 1.00 |
| T cell populations |  |  |
| Th1 D0 | 0.57 | 0.97 |
| **Th2 D0** | **0.01** | **1.07** |
| Th2/Th1 D0 | 0.58 | 1.01 |
| Treg D0 | 0.52 | 1.08 |
| Th1 D3 | 0.61 | 0.98 |
| **Th2 D3** | **0.001** | **1.11** |
| **Th2/Th1 D3** | **0.000** | **1.06** |
| Treg D3 | 0.14 | 0.69 |
| Th1 D7 | 0.08 | 0.91 |
| **Th2 D7** | **0.000** | **1.10** |
| **Th2/Th1 D7** | **0.000** | **1.70** |
| Treg D7 | 0.72 | 1.02 |
| Peripheral blood cell counts |  |  |
| WBC D0 | 0.41 | 1.03 |
| ALC D0 | 0.34 | 0.53 |
| WBC D3 | 0.05 | 1.08 |
| ALC D3 | 0.51 | 0.71 |
| **WBC D7** | **0.03** | **1.14** |
| **ALC D7** | **0.04** | **0.17** |
| Inflammatory indicators |  |  |
| PCT D0 | 0.92 | 1.003 |
| hs-CRP D0 | 0.61 | 1.00 |
| **PCT D3** | **0.038** | **1.075** |
| hs-CRP D3 | 0.69 | 1.00 |
| PCT D7 | 0.66 | 1.02 |
| hs-CRP D7 | 0.71 | 1.00 |

APACHE = Acute Physiology and Chronic Health Evaluation; SOFA = Sequential Organ Failure Assessment; Th = T helper; Treg = Regulatory T cells; WBC = White Blood Cell; ALC = Absolute Lymphocyte Count; PCT = Procalcitonin; hs-CRP = Hyper-sensitive C-reactive protein; Significant associations with p<0.05 are in bold.

**Table S2** Univariate cox regression analysis of variables associated with the 28-day mortality in non-pulmonary sepsis.

| **Variables** | **P** | **Exp(B)** |
| --- | --- | --- |
| Age | 0.30 | 1.049 |
| gender | 0.08 | 6.896 |
| Comorbidities |  |  |
| Hypertension | 0.18 | 4.541 |
| **Cardiovascular dysfunction** | **0.03** | **7.731** |
| Diabetes | 0.12 | 4.138 |
| Cerebrovascular disease | 0.96 | 0.940 |
| Chronic renal dysfunction | 1.00 | 1.000 |
| Severity score |  |  |
| APACHE II | 0.17 | 1.158 |
| SOFA D0 | 0.69 | 0.939 |
| SOFA D3 | 0.24 | 1.186 |
| SOFA D7 | 0.62 | 1.151 |
| T cell populations |  |  |
| **Th1 D0** | **0.05** | **0.802** |
| Th2 D0 | 0.46 | 1.030 |
| **Th2/Th1 D0** | **0.02** | **1.096** |
| Treg D0 | 0.06 | 1.200 |
| Th1 D3 | 0.11 | 0.861 |
| **Th2 D3** | **0.002** | **1.222** |
| **Th2/Th1 D3** | **0.003** | **2.896** |
| Treg D3 | 0.74 | 1.098 |
| Th1 D7 | 0.14 | 0.890 |
| **Th2 D7** | **0.04** | **1.253** |
| Th2/Th1 D7 | 0.08 | 5.021 |
| Treg D7 | 0.82 | 0.865 |
| Peripheral blood cell counts |  |  |
| WBC D0 | 0.11 | 1.102 |
| ALC D0 | 0.99 | 0.986 |
| **WBC D3** | **0.007** | **1.248** |
| ALC D3 | 0.15 | 0.221 |
| **WBC D7** | **0.019** | **1.487** |
| ALC D7 | 0.18 | 0.027 |
| Inflammatory indicators |  |  |
| PCT D0 | 0.29 | 0.949 |
| hs-CRP D0 | 0.24 | 0.621 |
| PCT D3 | 0.40 | 0.703 |
| hs-CRP D3 | 0.90 | 1.001 |
| PCT D7 | 0.61 | 0.813 |
| hs-CRP D7 | 0.45 | 0.975 |

APACHE = Acute Physiology and Chronic Health Evaluation; SOFA = Sequential Organ Failure Assessment; Th = T helper; Treg = regulatory T cells; WBC = White Blood Cell; ALC = Absolute Lymphocyte Count; PCT = procalcitonin; hs-CRP = Hyper-sensitive C-reactive protein; Significant associations with p<0.05 are in bold.

**Table S3** The power calculation of the parameter with detected difference between pulmonary and non-pulmonary sepsis with α=0.10

| Parameter | Power | β |
| --- | --- | --- |
| Th1 on D0 | 0.71 | 0.29 |
| Th1 on D3 | 0.99 | 0.01 |
| Th1 on D7 | 0.54 | 0.45 |
| Th2 on D7 | 0.86 | 0.14 |
| Th2/Th1 on D3 | 0.75 | 0.25 |
| Th2/Th1 on D7 | 0.83 | 0.17 |

Th = T helper.


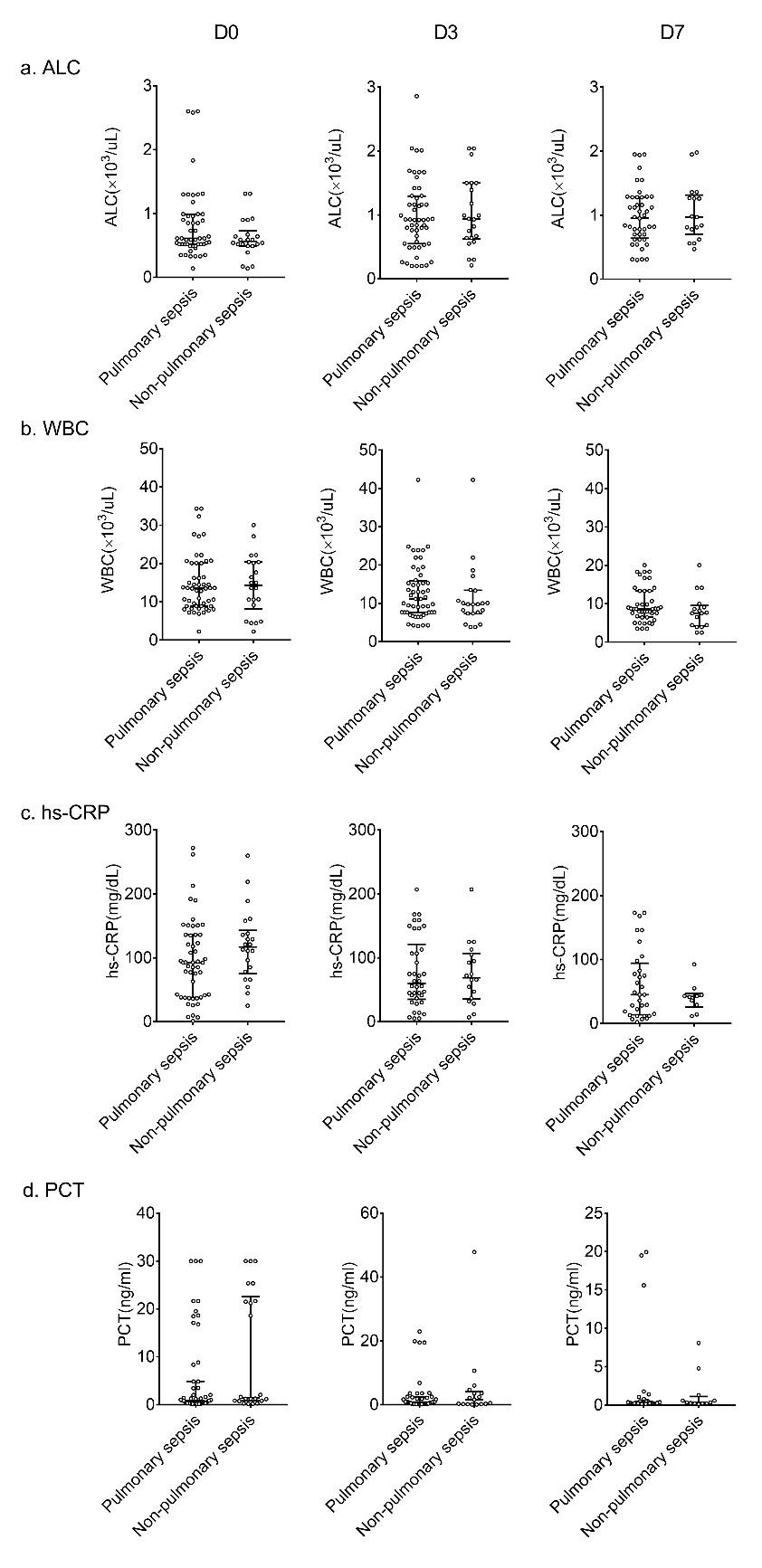


**Figure S1** Inﬂammatory and immune indicators in pulmonary and non-pulmonary sepsis. Data regarding to ALC (**a**), WBC (**b**), hs-CRP (**c**) and PCT (**d**) was shown with scatter dot plots and lines of median with interquartile range. Comparisons were determined by using Mann-Whitney U test.
